# Characterization of Selected Plant Growth-Promoting Rhizobacteria and Their Non-Host Growth Promotion Effects

**DOI:** 10.1128/spectrum.00279-21

**Published:** 2021-06-30

**Authors:** Di Fan, Donald L. Smith

**Affiliations:** a Department of Biological and Environmental Engineering, School of Biology, Food and Environment, Hefei University, Hefei, China; b Department of Plant Science, McGill University, Macdonald Campus, Sainte-Anne-de-Bellevue, Quebec, Canada; University of Minnesota

**Keywords:** biofertilizers, plant growth promotion rhizobacteria, *Arabidopsis*, induced systemic resistance, maize

## Abstract

Plant growth-promoting rhizobacteria (PGPR) are a functionally diverse group of microbes having immense potential as biostimulants and biopesticides. We isolated four PGPR (designated n, L, K, and Y) that confer growth-promoting effects on Arabidopsis thaliana. The present study describes the detailed polyphasic characterization of these PGPR. Classical methods of bacterial identification and biochemical test kits (API20E, API20NE, API ZYM, and API 50CH) revealed their metabolic versatility. All rhizobacterial isolates were positive for 1-aminocyclopropane-1-carboxylate (ACC) deaminase (ACCD) and indole acetic acid production and phosphorous solubilization. PCR analysis confirmed the presence of the *nifH* gene in strains n, L, and Y, showing their N_2_-fixation potential. *In vitro* dual culture methods and bacterial infestation *in planta* demonstrated that strains n and L exerted antagonistic effects on Pseudomonas syringae pv. tomato DC3000 and *Botrytis cinerea* 191 and provided protection to *Arabidopsis* plants against both phytopathogens. Short- or long-term bacterial treatment revealed significant changes in transcript levels of genes annotated to stress response and hormone metabolism in *A. thaliana*. In particular, the expression of stress-responsive genes in *A. thaliana* showed an upregulation under salinity stress. MAP kinase 6 (MPK6) was involved in the growth promotion induced by the four bacterial strains. Furthermore, these strains caused a significant increase in root dry weight of maize seedlings under gnotobiotic conditions. We conclude that the four rhizobacteria are good candidates as biofertilizers for enhancing growth of maize, among which strains n and L showed marked plant growth-promoting attributes and the potential to be exploited as functional biostimulants and biopesticides for sustainable agriculture.

**IMPORTANCE** There are pressing needs to reduce the use of agrochemicals, and PGPR are receiving increasing interest in plant growth promotion and disease protection. This study follows up our previous report that the four newly isolated rhizobacteria promote the growth of Arabidopsis thaliana. We test the hypothesis that they have multiple PGP traits and that they can be used as biofertilizers and biopesticides. *In vitro* assays indicated that these four strains have various PGP properties related to nutrient availability, stress resistance, and/or pest organism antagonism. They significantly influenced the transcript levels of genes involved in stress response and hormone metabolism in *A. thaliana*. MPK6 is indispensable to the growth stimulation effects. Strains n and L protected *A. thaliana* seedlings against phytopathogens. Three strains significantly increased maize growth *in vitro*. In summary, introducing these four strains onto plant roots provides a benefit to the plants. This is the first study regarding the potential mechanism(s) applied by *Mucilaginibacter* sp. as biostimulants.

## INTRODUCTION

A close association between microbes and terrestrial plants is thought to have existed for approximately half a billion years. The plant-associated microbiome has now been recognized as the phytomicrobiome, containing a diversity of collaborating and competing species that are essential to the well-being of their host plants (1). Plants and rhizospheric microbes (in the rhizosphere or on the root surface) and endophytic microbes (inside plant tissues) have complex associations with each other, and these are critical for both plants and microbes in terms of nutrient uptake, survival, development, growth, and reproduction. Root-colonizing beneficial bacteria, referred to as plant growth-promoting rhizobacteria (PGPR), are known to include species of Pseudomonas, *Azospirillum*, *Azotobacter*, Klebsiella, Enterobacter, *Alcaligen*, *Arthobacter*, *Burkholderia*, *Bacillus*, and *Serratia*, which have been shown to assist plant growth and to control plant diseases (2, 3), either directly or indirectly, through nitrogen fixation (4), antagonism to phytopathogens (5), improving plant responses to abiotic stressors (6), alteration/production of phytohormones, and soil nutrient mobilization (7). Recently, some additional PGP traits have been discovered, such as bacteriocin production, production of microbe-to-plant signals (8), and sulfur deficiency alleviation (9); it seems likely additional mechanisms of plant growth promotion will be revealed as these relationships are further explored. *Bacillus* and fluorescent Pseudomonas spp. are the most studied and exploited bacteria as biocontrol agents (10, 11).

Plants have mechanisms to cope with various environmental stresses, the most common being soil salinity, low temperature, and drought, all of which can elicit common gene responses related to nearly every aspect of plant morphology, physiology, and metabolism (12), leading to inhibition of seed germination, seedling growth, flowering, and seed set (13). Soil salinity adversely affects plant growth via both osmotic and ionic stresses and has become a major limiting factor in agricultural production worldwide, leading to as much as $27 billion in losses per year (14). Salinity stress disrupts the cellular osmotic balance by lowering the water potential inside cells (15). Stressfully high salt conditions subsequently induce oxidative stresses by generating reactive oxygen species (ROS) (16) within cells, resulting in oxidative damage of membrane lipids, proteins, and nucleic acids (17). To cope with salt stress, stressed plants activate various mechanisms through conserved signal transduction pathways (18), resulting in the production and accumulation of diverse functional components, such as osmolytes (i.e., proline and glycine betaine) (19) and nonenzymatic (i.e., phenolics, flavonoids, and glutathione) and enzymatic (i.e., peroxidase, catalase, and the enzymes involved in the ascorbate-glutathione cycle) (20, 21) antioxidants, all of which mitigate the oxidative damage caused by high salinity (18).

There have been numerous reports of PGPR-mediated salt tolerance in plants (22). For example, salt stress in tomato can be ameliorated by Achromobacter piechaudii ARV8, producing 1-aminocyclopropane-1-carboxylate (ACC) deaminase (ACCD) (23). Bacillus subtilis GB03 conferred salt tolerance via tissue-specific regulation of the ion transporter high-affinity K^+^ transporter 1 (HKT1) in *Arabidopsis* (24). In a recent report, a carotenoid producing halotolerant PGPR, *Dietzia natronolimnaes* STR1, was shown to boost salt tolerance in wheat (22). Mechanistically, such tolerance is explained by means of hormone homeostasis, production of ACCD and volatile compounds, stimulation of stress-related gene expression, and sodium uptake/transport (25). An ABA signaling cascade and innate immunity enhancement were mainly involved in the stress resistance (22, 26). Ethylene is important for plant growth and development, but excessive amounts of ethylene can decrease root growth (27). The production of ACCD is linked to stress resistance, because abiotic stressors increase ethylene production from ACC in plants (25, 28).

During the last few decades, chemical pesticides have been the main strategy to manage phytopathogens. However, like chemical fertilizers, the extensive use of chemical pesticides can lead to negative effects on the environment and food safety. Biopesticides are usually inherently less toxic, very targeted, and biodegradable, so they have generated enormous interest as promising alternatives to chemical control (29). Over the last several decades, a great diversity of rhizobacteria, such as species within the genera *Bacillus* and Pseudomonas, have been reported to have antipathogen activities and effectively ward off a broad spectrum of phytopathogens in plants (7, 30). The mechanisms employed by these bacteria for disease suppression in plants may be a function of their ability to control niche-space competition, nitrogen fixation, phosphate solubilization, and production of antibiotics, siderophores, volatile compounds, hydrolytic enzymes, and phytohormones (31). More recently, there have been clear demonstrations of specific signal compounds exchanged between plants and rhizosphere bacteria (members of the phytomicrobiome) that control each others’ gene expression, protein production, physiology, and growth (1). Moreover, rhizobacterium-induced systemic resistance (ISR) (32) has been reported in many plant species (i.e., rice, bean, cucumber, tobacco, tomato, and *Arabidopsis*) and is effective against a broad range of phytopathogens, including fungi, bacteria, viruses, and insect herbivores. *A prior* infection, such as microbial symbiosis, that is effective for a period of time can induce resistance to pathogenic attacks that occur after symbiosis establishment (33). ISR requires the plant defensin 1.2 (*PDF1.2*) gene and is largely mediated by jasmonic acid (JA) and ethylene (ET) signaling pathways (34). Previous studies reported that rhizobacterial strains trigger robust ISR against pathogens in various plant species via either JA/ET, ET, or salicylic acid (SA) signaling pathways or some combination of these (35–37). Beneficial rhizobacteria trigger ISR, thereby priming plants for the potentiated activation of various cellular defense responses, such as oxidative burst, cell wall reinforcement (38), defense-related enzyme accumulation, and phytoalexin production (39).

In our previous study, the four selected bacterial strains n, L, K, and Y, belonging to the genera Pseudomonas, *Bacillus*, *Mucilaginibacter*, and *Rhizobium*, respectively, were demonstrated to facilitate seedling growth and alleviate salt stress in *Arabidopsis* (40). As an effort to dissect the mechanism of the rhizobacterium-mediated growth promotion and stress amelioration, the major goals of the present work, with regard to the four isolates investigated, were to (i) characterize their physiological attributes; (ii) assess their PGP traits *in vitro*; (iii) assess their antagonistic ability against phytopathogens; (iv) investigate the possible PGP mechanisms of selected strains by using real-time quantitative PCR (RT-qPCR); and (v) evaluate their effects on early seedling growth of maize. To our knowledge, this is the first report on a *Mucilaginibacter* sp., in this case from wild dandelion (*Taraxacum officinale*) in Canada, that showed growth stimulation effects on a crop plant.

## RESULTS

### Selected bacterial strains possessed multiple PGP traits.

The four selected strains were thoroughly examined for several traits that are often associated with biocontrol and plant growth promotion.

The Nitrogen-free bromothymol blue malate (NFb) medium methodology revealed that strains n, L, and Y probably had nitrogen-fixing capacity in the solid culture conditions used, since the color of medium was changed from green to blue (see Fig. S1a in the supplemental material). For the PCR specific for the *nifH* gene, the expected fragment (390 bp) was obtained from strains n, L, and Y (Fig. S1b). The amplified fragments of *nifH* gene from strains n, L, and Y were deposited into NCBI GenBank, and their accession numbers are MW467562, MW467563, and MW475351, respectively.

The P solubilization ability was tested in NBRIP agar medium. Clear dissolution halos were observed around the colonies of strains n (Fig. S2a), L, and Y. Strain n (Pseudomonas sp.) was capable of greater levels of solubilization (5.67 mm) than strains L (*Bacillus* sp.) and Y (*Rhizobium* sp.) (Table 1). Since the buffering capacity of soil could limit the ability of bacteria to solubilize soil P, phosphate agar buffered with 100 mM Tris-HCl (pH 8.0) to mimic the buffering capacity of alkaline vertisols was chosen to ascertain the efficacy of PSB. Only strain n, a Pseudomonas strain, showed the diagnostic pink coloration zone (Fig. S2b). The quantity assay showed that strain n (Pseudomonas sp.) exerted significantly (*P < *0.001) greater P solubilization (maximized at 350 μg ml^−1^ on the 7th day of incubation), while strains L (*Bacillus* sp.) and Y (*Rhizobium* sp.) showed similar but much lower levels of activity, with the largest amount of solubilized P (69 and 63 μg ml^−1^) on the 14th day of incubation. Strain K showed very low activity.

**TABLE 1:** *In vitro* biochemical characterization of bacterial isolates on the plant growth promotion traits^*f*^

Strain	IAA concn^*a*^ (mg liter^−1^)	ACC deaminase activity^*a*^ (μmol mg^−1^ h^−1^)	P solubilization^*b*^ (mm)	Siderphores on CAS agar^*c*^	Nitrogen fixation	HCN production^*d*^	Ammonia production	Antimicrobial activity^*e*^	
								Pseudomonas syringae	*Botrytis cinerea*
**n**	0.79^a^	8.10^a^	5.67^a^	5	+++	+++	++	2.00^b^	5.00^a^
**L**	0.10^b^	2.01^b^	2.83^b^	1	+	−	+++	6.33^a^	2.67^b^
**K**	0.83^a^	0.98^c^	−	−	−	−	−	−	−
**Y**	0.86^a^	4.09^b^	2.17^b^	−	++	−	−	−	−

a Values are presented as means from 6 replicates.

b Means of diameter of solubilization measured on National Botanical Research Institute’s phosphate (NBRIP) agar plates (average of 6 replicates).

c Means of size of halo measured on chrome azurol S (CAS) plates (average of 6 replicates).

d HCN production was examined by change in color soaked in picric acid from yellow (control plate) to bright orange (strain n).

e Values are the mean diameters of inhibition zones (mm) (average of 4 replicates).

f Means followed by different letters within a row are significantly different (*P* ≤ 0.05). +, low activity; ++, moderate activity; +++, strong activity; −, no activity.

Auxin production *in vitro* by bacterial isolates was investigated colorimetrically as indole acetic acid (IAA) equivalents in the presence of l-tryptophan. Strain n produced an average of 0.79 μg ml^−1^ of IAA (or its intermediates), which was comparable to levels produced by strains K and Y (Table 1). The *Bacillus* sp. (strain L) produced significantly smaller amounts of IAA (0.1 μg ml^−1^) than strains n, K, and Y.

The ACC-deaminase activity in the four strains was determined quantitatively by monitoring the amount of ketobutyrate generated by hydrolysis of ACC. Strain n had maximum ACCD activity (8.10 μmol mg^−1^ h^−1^), while strain K exhibited the lowest ACCD activity (0.98 μmol mg^−1^ h^−1^) (Table 1). The production of ammonia, siderophores, and hydrogen cyanide (HCN) was also monitored. All strains, except for strain K, were capable of producing ammonia. Only strains n and L showed indications of hydroxamate type siderophore production on CAS plates. Strain n exhibited much higher levels of siderophore activity (+++) than strain L, which showed only a minor production of siderophore (+) (Table 1). Moreover, only strain n showed positive results for HCN production. Based on the intensity of orange color, the amount of ammonia produced from highest to lowest was strain L (+++), followed by strain n (++), and finally the *Rhizobium* strain Y (+).

Only strain L showed a clear hemolytic halo (beta-hemolysis) around the colony. Strain n showed alpha-hemolysis, while strains K and Y manifested γ-hemolysis. Antagonistic activity was assessed for both pathogenic bacterium and fungus. Both strains n and L showed growth suppression of *B. cinerea* B191, but the inhibition with strain n was more prominent than that with strain L (Table 1). Conversely, strains K and Y showed no inhibition effects compared with control plates (without rhizobacterial inoculation). Of the tested bacteria, strains n and L demonstrated growth antagonism toward Pseudomonas syringae DC3000, with inhibition zones (clear of pathogenic bacterial growth) of 2 and 6.33 mm, respectively (Table 1). Strains K and Y exhibited no antagonistic activity; in their presence, P. syringae DC3000 manifested confluent growth similar to that in control medium plates.

### Primary characterization of selected rhizobacteria.

All four strains were positive for catalase and negative for oxidase. Strains n, K, and Y were found to be Gram negative and positive for Voges-Proskauer (VP) reaction (acetoin production), while strain L was Gram positive and showed positive tests for nitrate reductase, amylase, and gelatinase. Only strains L and Y were motile. All four of the strains grew well on KB, LB, tryptic soy agar (TSA), and R2A agar medium, but only strain n showed growth on MacConkey agar (all from Difco) with a pink colony color, indicating that strain n is a lactose-fermenting Pseudomonas. Only strains K and Y showed a mucoid phenotype on RVC-agar medium, indicating potential exopolysaccharide (EPS) production by the two isolates. All these morphological and biochemical characteristics are presented in Table S2.

The selected isolates were also tested for extracellular enzymatic profiles involved in the breakdown of peptides, phosphomonoesters, lipids, mucopolysaccharides, polysaccharides, chitin, cellulose, starch, and galactans (Table S2). All four strains exhibited strong leucine arylamidase activity. The remaining tests produced differentiating patterns among the four genera examined.

Tolerance of abiotic stressors by the four strains was also studied. High concentrations of NaCl repressed bacterial growth; strains n and L tolerated higher concentrations of NaCl than K and Y (Table S2). Strains L and K can tolerate up to 40 and 33°C, respectively. All the strains grew well at pHs 5, 6, 7, and 8, but only strains n and L were able to grow at pHs 9 and 10. Overall, strain L was the most tolerant to these stressors.

The selected strains used a large variety of carbohydrates as sole carbon and energy sources (Table S3). All strains acidified fructose. All except for strain n were found to hydrolyze esculin, while only strain L was negative for citrate and malonate utilization. Only strain K produced acid from raffinose. Thus, in total, strain n, followed by L, showed utilization of the broadest range of carbon sources (58% of those tested) (Table S3).

Data in Table S4 indicated that strains n, L, and Y were highly sensitive to kanamycin while strain K was very resistant (up to 900 μg ml^−1^). The same trend occurred for tobramycin, gentamicin, and tetracycline. With regard to ampicillin, strain Y was very sensitive, while the other bacteria showed strong resistance (>256 μg ml^−1^). For erythromycin and chloramphenicol, strain n showed greater resistance than strain K, while strains n and Y were susceptible to them. For vancomycin, strains n and Y demonstrated substantial resistance, while strains L and K were sensitive. Overall, there was a mixed pattern observed for the tested antibiotics (Table S4).

### Selected strains alleviated disease severity in *Arabidopsis*.

**FIG 1:**
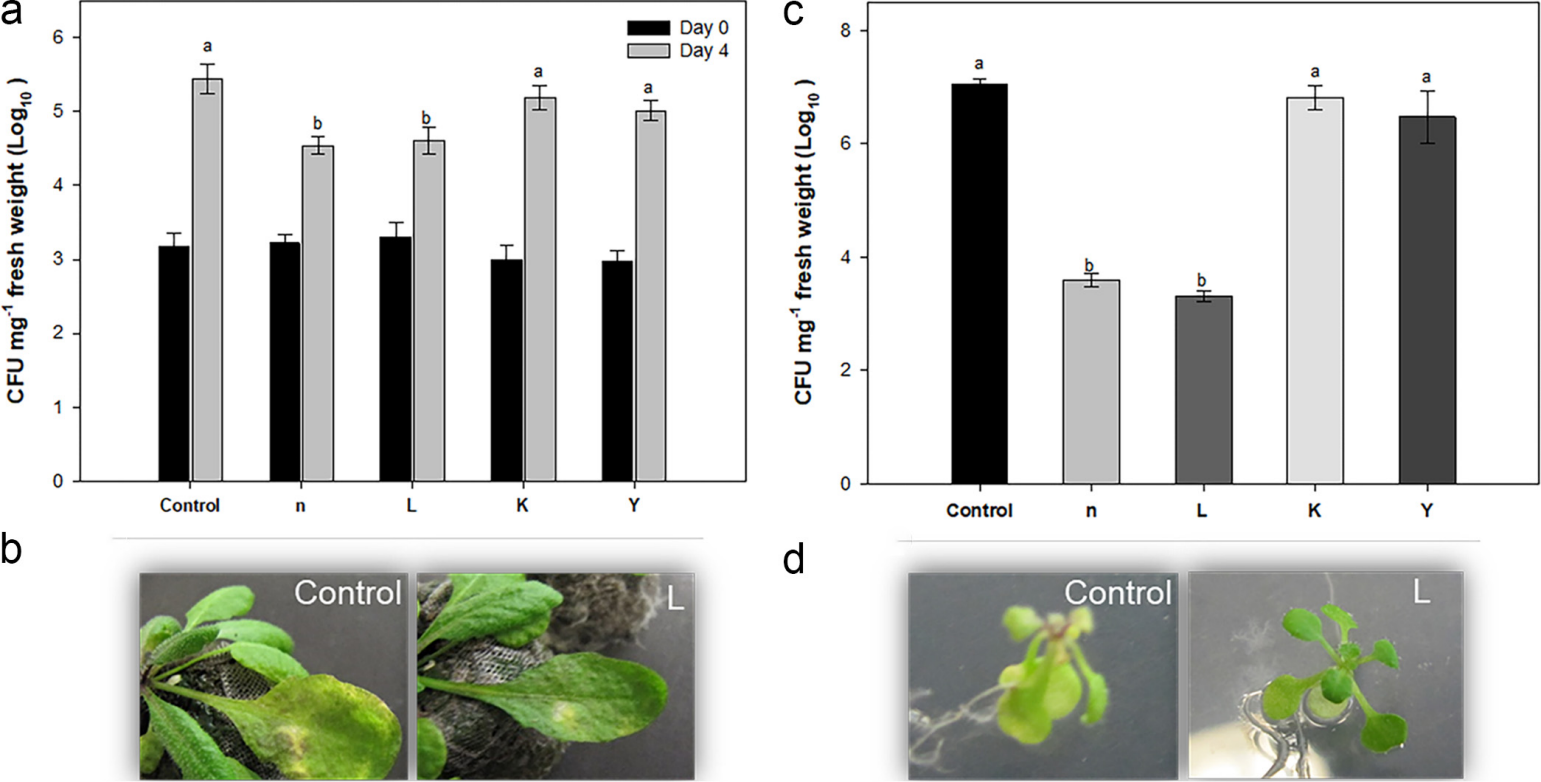
Effects of treatment of *Arabidopsis* wild-type Col-0 with isolated rhizobacteria on resistance against a bacterial pathogen. In the *in planta* experiment, 4-week-old plants were challenge infected with P. syringae pv. tomato DC3000. (a and b) The growth of P. syringae pv. tomato DC3000 (a) and disease symptoms (b) in control and rhizobacterium-treated plants were investigated. (c and d) An *in vitro* experiment showed significant reduction in disease symptoms in seedlings treated with rhizobacteria (d), as confirmed by the inhibition of P. syringae pv. tomato growth (c). The number of bacteria in the leaves was determined at 7 days after drop inoculation with a suspension at 10^9^ CFU ml^−1^ in a plate assay. Different letters indicate statistically significant differences among treatments (*P < *0.01). Bars indicate means ± standard errors from three independent experiments, with 12 inoculated leaves per treatment.

Leaves of 4-week-old *Arabidopsis* plants were challenged with P. syringae pv. tomato DC3000 and showed typical yellowing symptoms on the 3rd day after inoculation. Visual observations showed that plants treated with strains n and L strongly restricted disease spread compared to other treatments (Fig. 1a and b). Pathogenic bacteria colonizing *Arabidopsis* leaves were extracted from surface-sterilized leaves 0 or 4 days after inoculation. Extraction of P. syringae pv. tomato DC3000 immediately after infiltration resulted in approximately 3 × 10^3^ CFU mg^−1^ in both control and treated plants. Compared with immediate extraction, the number of virulent bacteria from control leaves and those treated with strains K and Y was increased from 3 × 10^3^ to 5.5 × 10^6^ mg^−1^, whereas treatment with strains n and L significantly inhibited the growth of P. syringae pv. tomato to 4.3 × 10^4^ mg^−1^. Significant reductions in P. syringae pv. tomato disease severity were also observed for *Arabidopsis* seedlings grown *in vitro* on one-half-strength Murashige and Skoog (1/2 MS) agar plates under the same bacterial treatments (Fig. 1c and d). Consistent with this macroscopic observation, the population density of P. syringae pv. tomato in *Arabidopsis* rosettes was reduced significantly for seedlings treated with either strain n or L.

**FIG 2:**
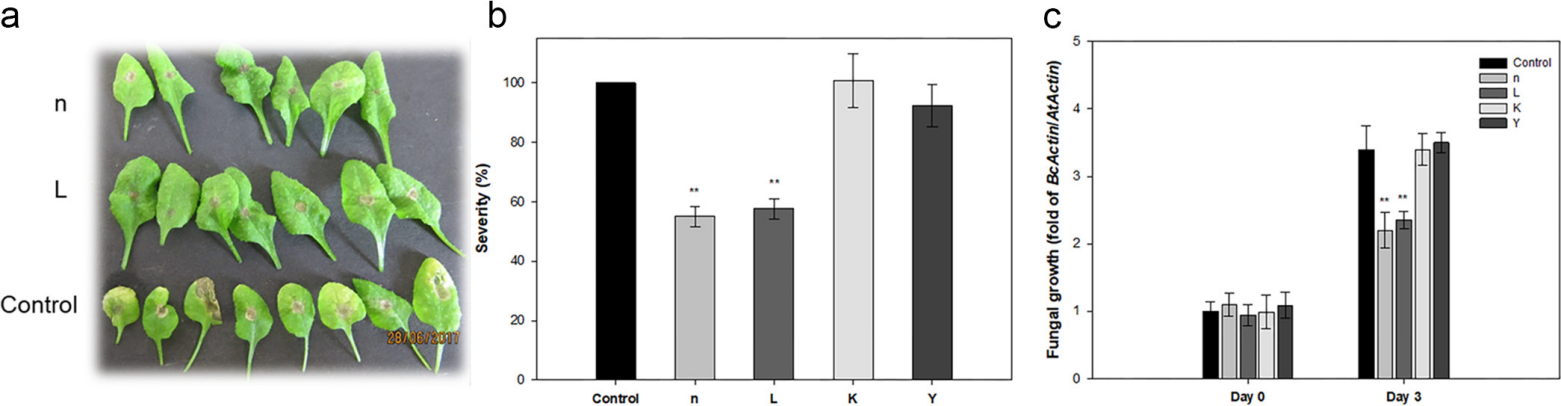
Effects of treatment of *Arabidopsis* wild-type Col-0 with isolated rhizobacteria on resistance against *Botrytis cinerea*. Four-week-old plants were challenge inoculated with spores of *B. cinerea* at 10^5^ CFU ml^−1^. (a) Visual comparison of disease symptoms on *Arabidopsis* leaves 3 days after inoculation. (b) At 7 days postinoculation, the average diameter of the expanding lesions formed in *Arabidopsis* leaves was measured. Disease severity, measured as the lesion diameter in the rhizobacterial treatments, was expressed as a percentage of the lesion diameter in the control treatments (set at 100%). (c) *In planta* growth of *B. cinerea*, as measured by simultaneous quantification of the expression levels of *B. cinerea Actin* gene (*BcActin*) and the *Arabidopsis Actin* gene (*AtActin*). Relative fungal growth was determined by ratios of *BcActin* to *AtActin*. Bars indicate means ± standard errors from three independent experiments, with 12 inoculated leaves per treatment (**, *P < *0.01 versus control).

*Botrytis cinerea* caused typical symptoms, such as water-soaked lesions at the inoculation loci and leaf yellowing around the spotting site in control leaves 2 days after infection. The lesion diameter (in millimeters) was measured on the 4th day after inoculation. We observed that the fungus caused spreading disease necrotic lesions and tissue damage in the leaves of *Arabidopsis*. The disease severity was similar among the control plants and plants that were pretreated with strains K and Y, while strains n and L caused significant improvement in resistance to *B. cinerea* over untreated control plants (Fig. 2a). The lengths of lesions were significantly reduced in plants treated with strains n and L compared with other treatments (Fig. 2b). Moreover, fungal growth, which is used as an indicator of the severity of pathogen infection, was measured 3 days after inoculation by RT-qPCR. In line with the reduced disease symptoms, fungal growth was significantly inhibited in leaf tissue of plants treated with strains n and L, indicating these two strains effectively protected *Arabidopsis* from *B. cinerea* (Fig. 2c).

### Effect of rhizobacteria on *Arabidopsis* transcript levels.

To assess the molecular mechanisms underlying the phenotypic effects observed in plants inoculated with the four bacterial strains under normal conditions, the expression levels of several chosen genes were measured at 12 h (short term) and 21 days (long term) after inoculation. For testing the early transcriptional changes in salt-stressed *A. thaliana* plants, 14-day-old seedlings were transferred to fresh plates supplemented with 100 mM NaCl, and RNA extractions were performed 12 h after transplanting. For the genes that were only significantly influenced under salt stress conditions, only the results from salinity stress were shown.

**FIG 3:**
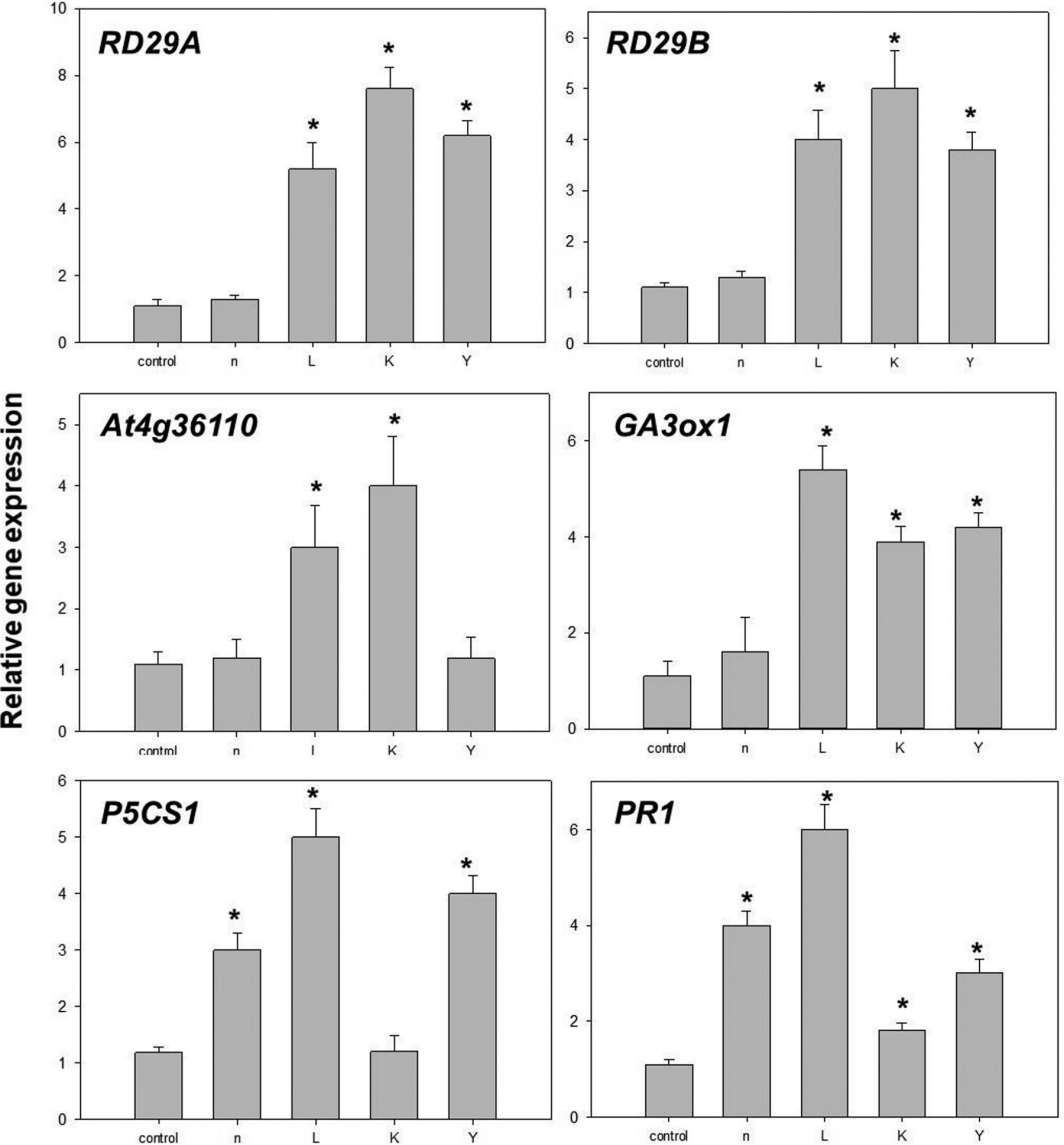
Quantitative measurements of the gene expression levels (fold differences) of six genes in *Arabidopsis* under salt stress. These genes were significantly influenced only under salinity conditions, not optimal growth conditions. Fourteen-day-old seedlings (seed-bioprimed) were treated with 200 mM NaCl. After 12 h, rosettes were collected and subjected to RNA isolation, following by quantitative real-time reverse transcription-PCR. Bars within the figure associated with an asterisk (*) represent values statistically different from the control at *P* < 0.05.

Twelve hours after imposition of salt stress, the expression levels of marker genes in ABA-dependent and/or independent pathways, *RD29A* and *RD29B*, were greater in seedlings treated with strains L, K, and Y than uninoculated controls; increases in the transcript levels of these two genes in bioprimed *A. thaliana* were salt stress dependent (Fig. 3). The expression of the *P5CS1* gene, which encodes rate-limiting steps in proline synthesis, was significantly upregulated in plants treated with strains n, L, and Y (Fig. 3). The same was true for *At4g36110* (SAUR-like auxin-responsive protein), whose transcriptional level was induced by strains L and K only under salt stress (Fig. 3). Inoculation with strains L, K, and Y resulted in a significant upregulation of *AtGA3ox1* (*Gibberellin 3-beta-dioxygenase*, At1g15550), which is one of the key genes regulating the synthesis of bioactive gibberellins.

**FIG 4:**
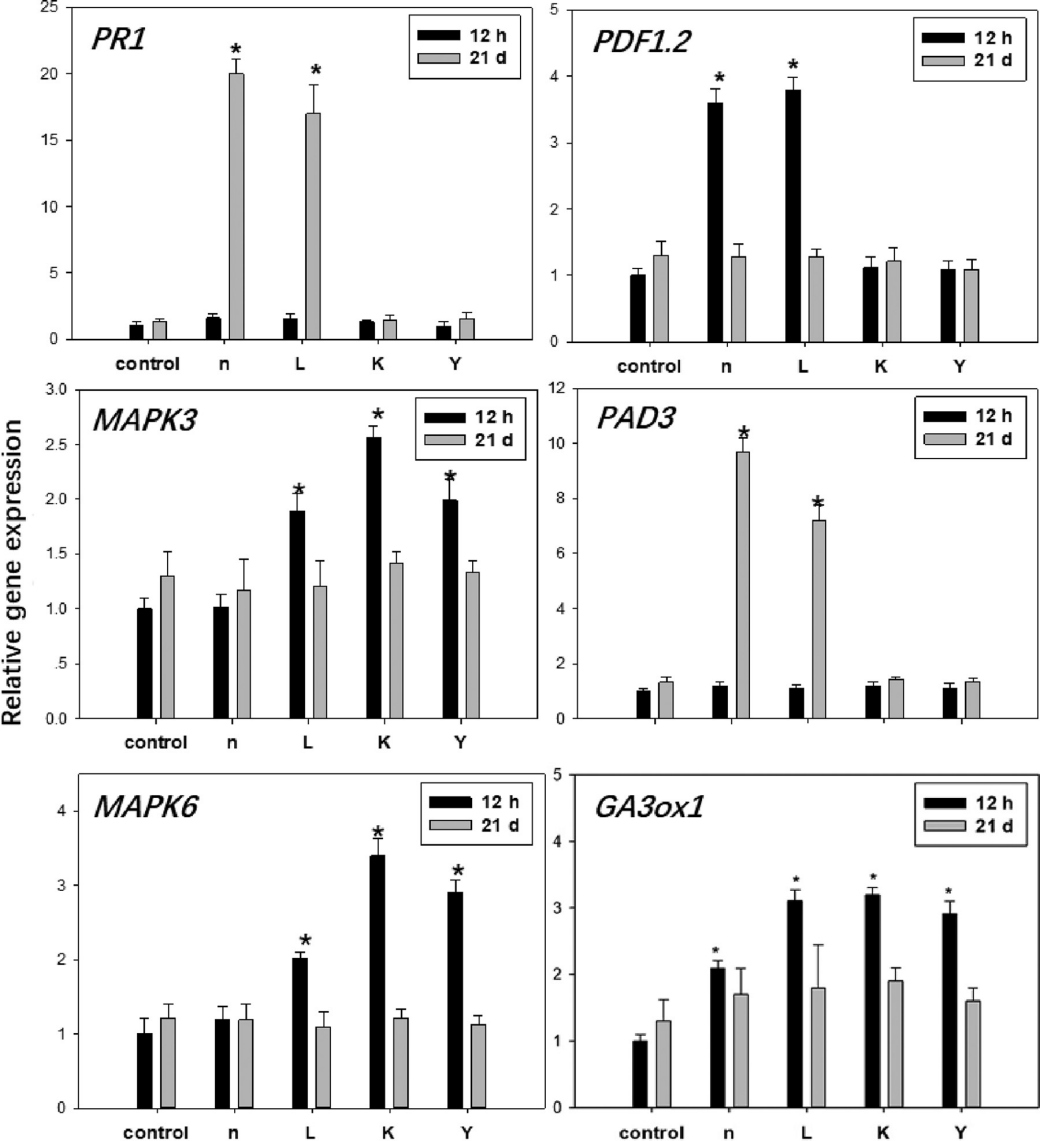
Quantitative measurements of the gene expression levels (fold differences) in *Arabidopsis* rosettes under normal growth conditions after bacterial treatment at 21 days and 12 h. Bars within the figure associated with an asterisk (*) represent values statistically different from the control at *P* < 0.05.

The effect of PGPR on plant defense priming was also investigated. The expression of *PR-1*, which is a salicylic acid (SA)-induced marker, showed 20- and 17-fold increases following treatment with strains n and L (long term), respectively (Fig. 4). Interestingly, under salt stress, plants inoculated with strains n, L, and Y showed a clear upregulation of *PR-1*; a minor but significant effect was also exerted by strain K inoculation (Fig. 4). *PDF1.2* gene expression was induced by strains n and L, with 2.6- and 4.8-fold increases, respectively, at 12 h postinoculation (Fig. 4). We also examined the expression levels of *MPK3* and *MPK6*. The transcript levels of the two genes were marginally, but significantly, increased by strains L, K, and Y at 12 h postinoculation (Fig. 4). However, salt treatment did not induce any differences between bioprimed and uninoculated conditions, at least within the first 12 h. At 21 days after inoculation with strains n and L, the transcript level of the camalexin synthetase gene *PAD3* was significantly higher than that in the control plants (Fig. 4).

**FIG 5:**
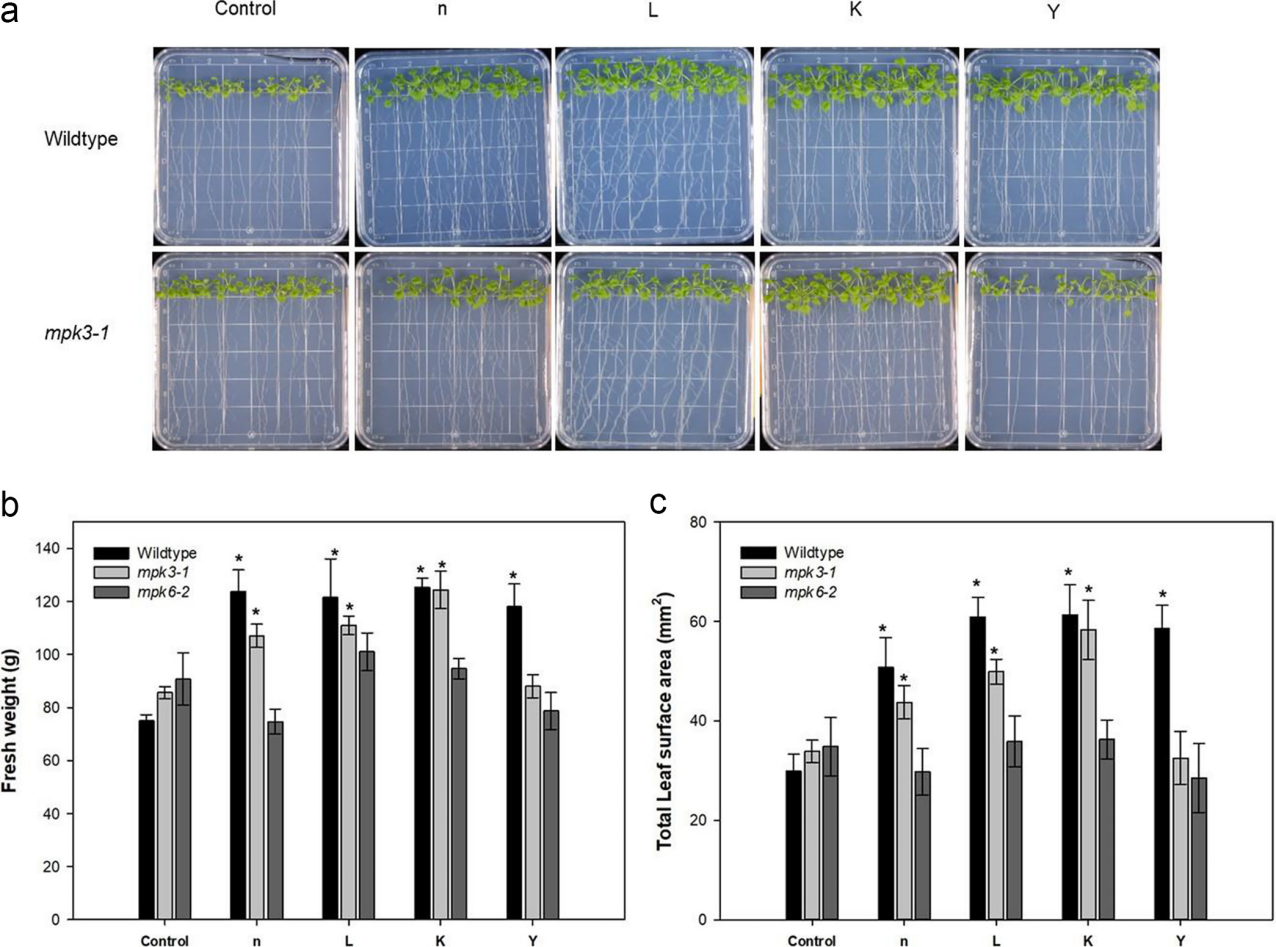
Effect of MPK3 and MPK6 mutants on the growth and morphometric parameters of *Arabidopsis* induced by inoculation of selected rhizobacterial strains. (a to c) Effect of knockout mutants on the phenotype (a), fresh weight (b), and total leaf surface area (c) of inoculated *Arabidopsis* for 21 days. Ten plants were pooled for each replicate for fresh and dry weight. Bars indicate means ± standard errors from six replicates (*, *P < *0.05).

The growth promotion effect was not hampered in *mpk3-1* seedlings inoculated with the strains n, L, and K (Fig. 5a). Seedlings of *mpk3-1* had greater fresh weight and leaf area when inoculated with strains n, L, and K than the control at 21 days postinoculation (Fig. 5b and c). The deficiency in *MPK6* gene expression limited the growth promotion effect by all four strains (Fig. 5b and c).

### Rhizobacterial inoculation promoted growth of maize seedlings.

**FIG 6:**
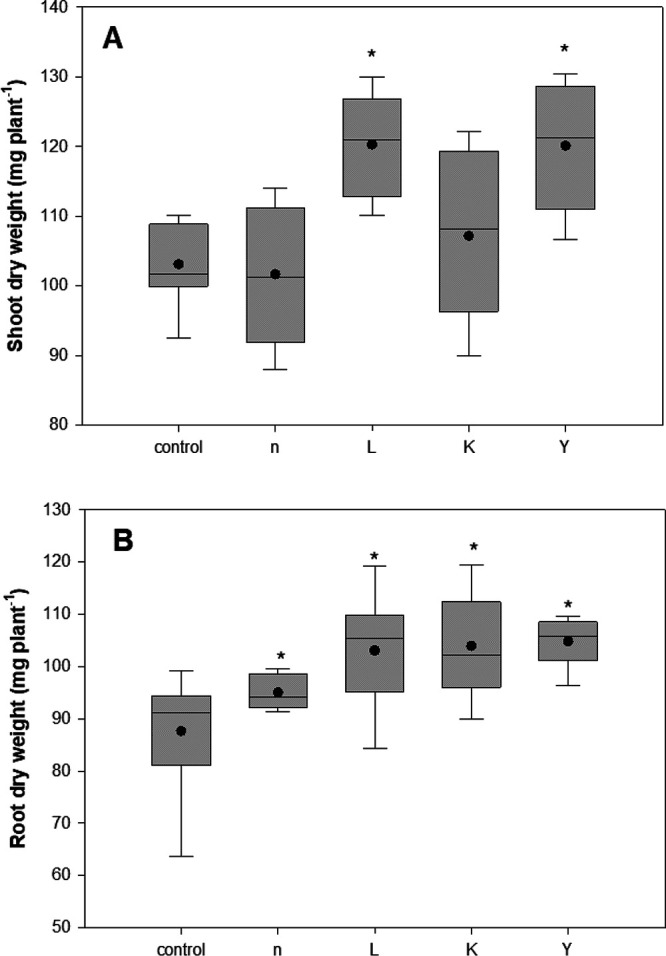
Boxplot for biomass (dry weight; mg seedling^−1^) of 21-day-old maize seedling seed inoculated with the four selected endophytic rhizobacterial strains under controlled conditions. The total range, interquartile range (boxes), and means (dots) are displayed. Asterisks indicate the statistically significant differences compared to controls (*, *P < *0.05).

Under unstressed conditions, maize seedlings inoculated with strains L and Y caused 1.6- and 1.8-fold increases in dry biomass of seedling shoots, respectively, along with significantly enhanced root dry weight due to inoculation of all four strains, over uninoculated controls (Fig. 6). Under salinity conditions (100 mM NaCl), seedlings inoculated with strains L and K showed a significant increase in the dry weight of shoot biomass (Fig. S3).

## DISCUSSION

Pseudomonads are among the most promising groups of rhizobacteria in terms of plant growth promotion, as they are aggressive colonizers (41) and usually manifest a wide range of PGP traits, such as antibiotic production, phosphate solubilization, nitrogen fixation, ACCD activity, production of plant-beneficial compounds (plant hormones, siderophores, EPS, IAA, HCN, and ammonia), and stress alleviation (42–44). *Bacillus* species/strains are omnipresent in nature, having immense potential for agricultural applications (45), as they can enhance crop yields by direct and indirect mechanisms, most of which are quite similar to those also seen in Pseudomonas (46). On the other hand, bacteria belonging to the genus *Rhizobium* are well-known for their ability to establish symbioses with both legumes and nonlegumes (47) and make atmospheric nitrogen available to the host plant (48). Species within the genus *Mucilaginibacter* are known to hydrolyze organic matter such as xylan and pectin and produce enormous amounts of extracellular polymeric substances (49). Although possessing various PGP traits, there are very few publications regarding their PGP effects (50). Madhaiyan et al. (51) reported that two novel *Mucilaginibacter* strains increased root length of tomato and canola seedlings in a gnotobiotic growth pouch assay. Thus, the PGP potential of the four selected rhizobacteria, belonging to the genera Pseudomonas, *Bacillus*, *Mucilaginibacter*, and *Rhizobium* (52), was examined.

Inoculation with rhizobacteria having ACCD activity resulted in enhanced root development (53), leading to better shoot growth (28). All four strains tested here showed ACCD activity, which were higher than the 20 nmol α-ketobutyrate mg^−1^ h^−1^ threshold (54), indicating that ACCD activity is involved in the mechanisms of plant growth stimulation by these strains. However, strains L and Y, with lower ACCD activity, caused greater root length enhancement in *A. thaliana* than other strains (40). The PGP effect was not strictly consistent with the ACCD activity level, indicating that ACCD activity is not a major PGP mechanism employed by these strains. All four strains produce IAA at levels ranging between 0.10 (strain L) and 0.86 mg liter^−1^, which is lower than some previous reports, demonstrating values of ca. 10 to 100 μg ml^−1^ for the same genus (55). Bacterium-produced IAA plays a multifaceted role in plant growth (56).

Outside the symbiotic interaction between legumes and alphaproteobacteria (57), nonspecific nitrogen-fixing bacteria also exist in relationships with a much wider range of plant species. Several Pseudomonas (58), *Bacillus* (59), and *Paenibacillus* (60) strains have been identified as nitrogen fixers. The results for nitrogen fixation, as confirmed by successful amplification of the *nifH* gene, a biomarker widely used to indicate nitrogen fixers (61), indicated that strains n, L, and Y are potentially nitrogen-fixing bacteria and may be beneficial in improving the nitrogen level of host plants.

Bacterium-mediated P solubilization is considered one of the most important PGP traits in rhizobacteria. In this study, we found that all four strains were positive for P solubilization, being able to convert insoluble tricalcium phosphate to the soluble forms in liquid medium, which is concordant with previous studies (49, 62). However, strains Y (*Rhizobium*) and L (*Bacillus*) solubilized much less P than indicated in previous reports for these genera. The maximum phosphate solubilization activity was detected in strain n (Pseudomonas sp.). Moreover, strain n could solubilize P in buffered medium, indicating that it is an efficient PSB, secreting strong or large amounts of organic acids, and may also perform well under soil conditions as a PSB.

Since siderophores aid in assimilation of iron, especially under iron-limited conditions, siderophore production ability represents biocontrol potential and plant growth promotion by strains n and L. A greenhouse experiment showed that strains n and L significantly increased plant growth for tomato (*Solanum lycopersicum* L.) under P and iron deficiency conditions, indicating that the traits of P solubilization and siderophore production are at least involved in growth stimulation of plants by these two strains (63).

Both HCN and ammonia are volatile compounds with biocontrol activity; they can function as biocontrol agents for suppression of plant diseases (64). Our results showed that only strain n, a Pseudomonas, produced HCN, while strains n and L could produce ammonia. More recently, Rijavec and Lapanje (65) suggested a novel role of HCN in increasing phosphate availability in plants, suggesting that strain n also deploys this mechanism for plant growth promotion. Hemolytic activity came to our attention because it is considered an indicator of activity on biological membranes, leading to potential antifungal activity of rhizobacteria (30). Thus, hemolytic activity has been associated with biocontrol of phytopathogens (66). In this study, only strain L was able to lyse red blood cells, indicating potential antimicrobial activity for this strain. Frikha-Gargouri et al. (67) demonstrated that the antibacterial activity of a *Bacillus* strain against plant-pathogenic Agrobacterium tumefaciens strains was correlated with hemolytic activity. Overall, strain n possessed the greatest number of possible PGP mechanisms.

In addition, strain L showed the most promising result with regard to antimicrobial activity against P. syringae pv. tomato DC3000, while for *B. cinerea*, strain n showed more encouraging abilities, similar to a previous report for Pseudomonas strains (68). Strains n and L, reported in this work, are siderophore producers and exhibit protease activity, and strain n produced HCN, all of which could be partially implicated in the antagonistic effects against phytopathogens. Ricci (69) reported that strains n and L showed clear antagonist abilities in response to three other phytopathogens, Clavibacter michiganensis, *Schlerotinia minor*, and Fusarium graminearum, suggesting the two strains have relatively broad spectra of biological activity and are promising potential biocontrol agents. PGPR have been shown to protect host plants from biotic stresses by provoking induced systemic resistance (ISR). Therefore, some of the present experimentation investigated the ability of strains n and L to prevent infection and to control the development of infections induced by *B. cinerea* and *P. syringe* tomato DC3000 in plant assays. Preinoculation of *Arabidopsis* with strains n and L resulted in significant reduction of disease symptoms in plants infested with P. syringae in both plate and pot experiments; in addition, they were effective for fungal resistance *in planta*, confirming the *in vitro* results and suggesting that pretreatment with isolated rhizobacteria n and L induced plant disease resistance against pathogens and reduced symptom development during disease susceptibility to *B. cinerea* and P. syringae pv. tomato DC3000, a possibly useful capability in competing against other rhizospheric microorganisms.

The four selected strains were also subjected to metabolic profiling. The traits of a bacterium associated with its fitness and metabolic assets contribute to a particular adaptation, providing support for its potential root colonization (70). The phenotypic patterns obtained revealed the substrate richness of these strains. The more metabolically flexible strains are generally more successful competitors in host plant colonization (71). Intriguingly, in this study, strains L and K were the most metabolically diverse of the four tested bacteria. Phenotypic profiling is of great importance for understanding genotype difference, stress responses, and environmental condition effects on rhizobacteria (72).

In general, PGPR with multifunctional traits are better than single traits (73); however, it is worth noting that, among the four selected strains, strain K possessed the fewest of the recognized PGP traits tested for but still caused significant growth promotion in *Arabidopsis* (40). It may have currently unrecognized PGP characteristics; for instance, it could be producing some currently unrecognized microbe-to-plant signal(s) (1). ACC-deaminase activity was expected to be one of the best indicators of effective PGPR under salinity stress, but our results showed that strain K had significantly lower ACCD activity than strain n, which had the highest level of ACCD activity, as well as IAA production among the four tested strains yet did not show stronger or comparable salt alleviation effects compared to the other three (40). Of the four tested bacteria, strains L, K, and Y were the best performers in the case of plant growth promotion and alleviation of negative salinity stress effects (40), while strains n and L possessed the greatest number of PGP traits, indicating factors other than common PGP traits must be employed in their plant growth-stimulating effects. Thus, it appears that rhizobacteria do not always have predictable effects on the growth and well-being of host plants with regard to currently recognized common PGP traits. This was also observed by Cardinale et al. (74). This interesting result suggested an explanation for the observation that a number of supposedly promising rhizobacterial strains selected from traditional PGP screening in fact eventually failed *in planta*, while the best candidates would have been discarded simply because of not performing well in *in vitro* PGP assays. It may be that some of these strains are producing currently unrecognized microbe-to-plant signals (1). The above-mentioned results have led to the conclusion that PGPR increased plant growth through a range of mechanisms, either by multiactivities of common PGP traits (74), through induced systematic resistance (75), or by untested PGP activities, such as sulfur oxidization (76), production of bacteria-to-plant signal molecules (i.e., oligosaccharides, peptides) (77), and, quite possibly, through other currently unrecognized PGP activities.

To further decipher the pathways that allowed enhanced plant vigor and induced defense machinery in *Arabidopsis*, molecular investigations were conducted using qPCR techniques. PGPR inoculation has been shown to change the expression pattern of genes in *Arabidopsis* plants; most of these genes are involved in signal transduction, defense response, and metabolism (78, 79). One of the PGP mechanisms utilized by PGPR is the induction of hormone production. Gibberellin hormones (GAs) can regulate almost every stage of growth and development in plants. *GA3ox1* (*At1g15550*), which is involved in GA biosynthesis and the determination of total leaf areas and flowering time (80), was upregulated by all four strains under normal growth conditions, and, in agreement, these plants produce greater leaf areas than uninoculated controls (40). A similar study has shown that *GA3ox1* gene expression was upregulated in 14-day-old *Arabidopsis* seeds treated with a PGPR, Burkholderia phytofirmans (81). In our study, the total number of leaves was not significantly affected by the four PGPR (data not shown), but the rosette area was (40), indicating that, at least under our experimental growth conditions and observation stage, these strains were promoting growth but not accelerating development. The study of the direct effect of these four rhizobacteria on plant metabolic rates, flowering time, and seeding are required to elucidate whether these bacteria accelerate plant metabolism and development.

The auxin-induced protein gene *At4g36110* was not affected by any of the four PGPR, indicating that the PGP activity of these bacteria was not caused by the production of IAA in plants under normal conditions. However, plants treated by strains L and K showed upregulated expression levels after salt exposure, indicating that the effect of salt stress alleviation by these two strains was caused by qualitatively altering endogenous IAA levels in *Arabidopsis*. It would provide more knowledge if the IAA content in different plant tissues were studied, particularly so if these studies included a range of plant growth conditions. A recent report showed that IAA content in wheat was increased by PGPR exposure under salt stress conditions (82). We observed that the expression level of *P5CS1* was not altered in plants under normal growth conditions but was augmented significantly 12 h after salt stress treatment in plants bioprimed by strains n, L, and Y. This gene is one of the rate-limiting factors in proline biosynthesis, and its transcription was associated with abiotic stress adaptation (83). Kim et al. (26) also reported that the expression of *P5CS1* was upregulated by PGPR in response to salt stress. Future study on the expression pattern of important ROS scavenging and ion homeostasis-related genes in *Arabidopsis* will open up new approaches allowing better understanding of the mechanisms involved in salt stress alleviation induced by the four PGPR studied.

Mitogen-activated protein kinase (MAPK) cascades in *Arabidopsis* have been shown to act as regulators of signaling pathways involved in immune responses (84) as well as biological processes, such as differentiation, proliferation, hormone signaling, osmotic stress, etc. (85, 86). MPK3 and MPK6 are two well-characterized signaling proteins in *Arabidopsis* (87). Rayapuram et al. (88) showed that although MPK3 and MPK6 have overlapping functions, they have differential preference for the downstream phospho-targets and are not replaceable by each other. Guo et al. (89) reported that MPK6 was involved in the leaf colonization and growth promotion induced by *Trichoderma vivide* Tv-1511 in *A. thaliana*. Our data showed that the accumulation of mRNA of *MPK3* and *MPK6* was slightly, but statistically, increased by short-term (12-h) PGPR treatment (strains L, K, and Y), which supported the hypothesis that bacterial biopriming induces the preactivation of stress-responsive machineries (38, 87). To explore whether *MPK3* and *MPK6* were target genes involved in the growth promotion effect of these strains, knockout mutants were used. Since *MPK6* mutants did not respond to all four tested strains in terms of growth promotion, we propose that the plant growth promotion effect was dependent of *MPK6* in all four tested strains of *Arabidopsis*. While *MPK3* was one of the target genes in the plant growth stimulation by strain Y, these results suggested the importance of contributions of *MPK6* and/or *MPK3* to the regulation of *Arabidopsis* growth promotion by the four tested rhizobacteria. More studies are needed to elucidate the potential upstream and/or downstream genes in *MAPK* signaling pathways that function in response to the application of these four strains in *Arabidopsis*.

Upon the initiation of the innate immune responses, plant cells trigger a series of signaling cascades that lead to diverse cellular responses, such as synthesis of the defense-related hormones (90), i.e., salicylic acid (SA), jamonic acid (JA), and ethylene (ET), and production of ROS. The expression of *PDF1.2* (encoding an ethylene and jasmonate-responsive plant defensin) and *PR1*, which are involved in JA and SA signaling pathways, respectively, were analyzed. Strains n and L significantly induced *PDF1.2* expression after 12 h of treatment. The highest gene expression of *PR1* was also achieved by treatment with strains n and L, but at day 21 after seed treatment. Interestingly, under salt stress, all four strains induced increases in *PDF1.2* expression by up to 6-fold (strain L). It has been reported that some PGPR did not affect *PR1* and *PDF1.2* expression under unstressed conditions (91). This is interesting in light of the recent demonstration that some key microbe-to-plant signals have meaningful effects only under plant stress conditions (92). Hong et al. (93) showed that the transcript level of both genes was induced by Paenibacillus polymyxa AC-1 after short-time exposure. It can be postulated here that strains n and L activate both JA and SA signaling pathways in *A. thaliana*, which is not an uncommon phenomenon (94), while SA signaling pathways were probably mainly associated with strains K and Y.

The expression of some stress response marker genes (*RD29A* and *RD29B*; related to abscisic acid signaling) that can be induced by salt and drought stress were also examined. Expression of these marker genes in bioprimed plants (with strains L, K, and Y) under salt stress conditions was significantly higher than those in control plants, suggesting that PGPR affect expression of these stress-related genes. This implied that exposure to PGPR that invoke biotic stress responses accelerates the activation of signaling machineries governing multiple stress adaptation processes.

Camalexin, 3-thiazol-2′-yl-indole, one of the major phytoalexins produced by *A. thaliana*, can be induced by both biotic and abiotic stress (95). Reduction in camalexin levels in *Arabidopsis* led to compromised resistance in the powdery mildew fungus *G. cichoracearum* (95). Here, we found that treatment with strains n and L led to significantly increased expression levels of *CYP71B15/PAD3* in *Arabidopsis* at day 21 (seed treated). This gene encodes a cytochrome P450 enzyme involved in the camalexin biosynthesis pathway. A microarray study by Mortel et al. (96) also showed that gene expression of *PAD3* was increased by Pseudomonas fluorescens SS101. This finding was in accordance with our results, reinforcing the possible involvement of camalexin in the biocontrol ability of strains n and L.

### Concluding remarks.

In this study, we investigated the four selected endophytic rhizobacteria from species of crop and uncultivated plants. The characterization study of these strains revealed their substantial potential in agriculture. Strains n and L performed well at biocontrol. The increased expression of genes related to hormone signaling and osmolyte production suggested an induction of systemic response in plants. Mutant plants with impaired hormonal pathways could be applied to confirm their involvement in plant growth stimulation and stress resistance. Future experiments that analyze spatial expression patterns of these genes are needed to properly explain the phenotypes observed in the present study. It would also be interesting to conduct transcriptome analysis in bioprimed *A. thaliana* after biotic or abiotic challenges to see whether there is primed ISR-specific gene expression. Moreover, inoculation with the four selected strains resulted in significant seedling growth promotion effects in maize (non-host). Thus, all four strains in this study are promising candidates to be developed as commercial biofertilizer formulations (strains n, L, K, and Y) as well as biopesticides (strains n and L) for agricultural production. Based on the phenotypic characteristics of selected isolates, strains n and L can tolerate extreme environmental conditions (high salinity) and possess the wide range of PGP traits tested, which differentiates them from the other two bacteria. In addition, the *nifH* gene amplification result confirmed that they are potential nitrogen fixers. This has important implications for biofuel production, where reducing inputs, such as costly nitrogen fertilizer, is highly desirable for production of biomass crops on more marginal lands. These two strains could be applied as inocula that may improve nitrogen fixation and lead to increased plant biomass production. Further studies should be focused on the potential of these four PGPR, as well as their coinoculants (63), to enhance plant growth in crops and for practical applications at the field level.

## MATERIALS AND METHODS

### Bacterial strains and plant materials.

The four rhizobacterial strains, designated n, L, K, and Y, were isolated from surface-sterilized root tissues of *Phalaris arundinacea*, *Solanum dulcamara*, *Scorzoneroides autumnalis*, and Glycine max, respectively, from Sainte-Anne-de-Bellevue, Québec, Canada (52). The strains were maintained on King’s B medium (KB; per liter double-distilled water [ddH_2_O], 20 g proteose peptone no. 3, 1.5 g K_2_HPO_4_, 1.0 g MgSO_4_, 10 ml glycerol, pH 7.2). The standardized bacterial suspensions were prepared in 10 mM MgSO_4_ according to Fan et al. (40) and were used for pathogen antagonism evaluation and bacterization experiments.

The *Arabidopsis MPK3* and *MPK6* T-DNA insertional mutants, referred to as *mpk3-1* (SALK_151594) and *mpk6-2* (SALK_073907), were provided by Fangwen Bai (Institut de Recherche en Biologie Végétale, Université de Montréal). Arabidopsis thaliana ecotype Col-0 plants and mutants were seed treated with bacterial suspensions and grown in petri dishes according to Fan et al. (40) for 3 weeks, after which the seedling pictures and fresh weight were taken. The total leaf surface area was quantified using ImageJ (http://rsb.info.nih.gov/ij).

### *In vitro* assays for PGP traits.

The four selected rhizobacterial strains were assessed for general PGP traits: (i) ammonia production; (ii) ACC deaminase activity; (iii) inodole-3-acetic acid (IAA) levels; (iv) nitrogen fixing ability; (v) phosphate solubilization; (vi) siderophore production; (vii) hydrogen cyanide production; (viii) antimicrobial activities; (ix) exopolysaccharide (EPS) production; and (x) blood hemolytic assay.

Ammonia production was detected using a Nessler’s reagent method (97). Quantitative measurement of ACC deaminase activity was estimated by measuring the amount of α-ketobutyrate produced from the cleavage of ACC as described by Penrose and Glick (54). For cyanogenic potential, bacteria were streaked on lysogeny broth (LB; per liter ddH_2_O, 10.0 g tryptone, 5.0 g yeast extract, 5.0 g NaCl, pH 7.0) agar medium supplemented with glycine (4.4 g liter^−1^), which serves as a precursor molecule (98). The production of auxin was quantitatively assayed (99) by culturing bacteria in KB medium supplemented with l-tryptophan (500 μg ml^−1^). Siderophore production was qualitatively assayed using the chrome azurol S (CAS) method (100). A clear halo around the colony with a color change from blue to orange on CAS agar medium indicates siderophore production.

The tricalcium phosphate [Ca_3_(PO_4_)_2_] solubilizing ability of bacterial strains was first tested on the National Botanical Research Institute's phosphate (NBRIP) growth medium (101). Mineral phosphate solubilizing ability was also evaluated under buffering conditions, using Tris phosphate medium (102) with 0.01% methyl red as a pH indicator. The phosphate-solubilizing potential then was quantitatively estimated according to Halder et al. (103).

Nitrogen-free bromothymol blue malate (NFb) medium (104) was used for visual detection of nitrogen-fixing activities of bacterial strains. To test for the presence of the *nifH* gene, the most widely utilized marker for nitrogen fixation (61), PCR was attempted using universal primers 19F (GCIWTYTAYGGIAARGGIGG) and 388R (AAICCRCCRCAIACIACRTC) (105). The PCR products were purified using a QIAquick gel extraction kit (Qiagen), cloned into the vector pDONR/Zeo (Invitrogen) using Clonase Gateway BP clonase II enzyme mix (Invitrogen), and then subjected to sequencing by the Genome Quebec Innovation Centre (McGill University, Canada). The amplified fragments of the *nifH* gene were then analyzed and identified by BLASTN program.

The antagonistic abilities of bacterial isolates were evaluated using a dual culture method, as described by Shehata et al. (106). Pseudomonas syringae pv. tomato DC3000 EV was maintained on LB agar plates supplemented with kanamycin (50 μg ml^−1^) and rifampin (10 μg ml^−1^) at 28°C. *Botrytis cinerea* wild-type isolate B191 was maintained on potato dextrose agar (PDA) amended with streptomycin (20 mg liter^−1^) at 22°C. For antibacterial assay, KB agar plates were inoculated with P. syringae pv. tomato DC3000 (10^8^ CFU ml^−1^) in sterile ddH_2_O prepared from overnight culture. Wells were then punched with a cork borer (5-mm diameter), and 50 μl of fresh cultures of each strain (10^8^ CFU ml^−1^) was applied to each well. All plates were then incubated at 28°C for 3 days to measure the diameter of the inhibition zones. For antifungal assay, a 5-mm agar plug from the colony margin of a 7-day-old PDA culture of *Botrytis cinerea* 191 at 22°C was recuperated and deposited in the center of a fresh PDA plate, with a 5-mm well filled with 50 μl of overnight culture of bacterial isolate approximately 2 cm away. Plates were incubated at 22°C and examined daily for antagonism by measuring the distance between the edges of the bacterial isolates and the fungal mycelium (zone of inhibition).

EPS production was detected macroscopically on RCV medium according to Santaella et al. (107). The possible activity on biological membranes via *in vitro* hemolysis assay was conducted according to Liu (108), in which each bacterium colony was transferred to blood agar plates (sheep blood plates; MP1301; HiMedia). Hemolysis was visualized by the development of a clear hemolytic halo (beta-hemolysis) around the colonies, indicating biosurfactant production. Detection of alpha-hemolysis was indicated by the production of greenish and dark color in the agar under the colony, which is usually caused by hydrogen peroxide produced by the bacterium, indicating partial damage of erythrocytes; γ-henolysis, or so-called nonhemolysis, resulted in no alteration of color or opacity in the agar medium.

### Quantitative measurement of gene expression via RT-qPCR analysis.

Arabidopsis thaliana wild type (Col-0) was seed treated and grown on petri dishes according to Fan et al. (40). Total RNA was extracted using a RNeasy plant minikit (Qiagen) by following the manufacturer’s recommendations. For each treatment, 15 plants were randomly withdrawn (rosettes from 5 plants were pooled for each of 3 replicates). The quality and quantity of the recovered total RNA were estimated by agarose (1%, wt/vol) gel runs and a NanoDrop ND-1000 spectrophotometer (NanoDrop Technologies, Inc.), respectively. Genomic DNA was removed, and first-strand cDNA was synthesized from 10 μg of total RNA using the QuantiTect reverse transcription kit (Qiagen) according to the manufacturer’s instructions. Expression analysis of key genes involved in hormone signaling and production, ISR, phytoalexin, and osmolyte production were performed on a CFX Connect real-time system (Bio-Rad) using SYBR green Ssoadvanced supermix (Bio-Rad) according to manufacturer recommendations. Each reaction was performed on 5 ng of the first cDNA strands. Gene-specific primers used are reported in Table S1 in the supplemental material. The expression level of target genes was normalized to *SAND* (*At2g28390*) in each RNA preparation and calculated using the 2^−ΔΔ^*^CT^* method (109). This experiment was repeated twice, with three biological and three technical replicates each time.

### Biochemical and physiological characterization of bacterial isolates.

The antimicrobial gradient diffusion method, using Etest (bioMérieux, La Balme-les-Grottes, France), was carried out to quantitatively determine the susceptibility of isolated bacteria to various antibiotics. Bacterial suspension (10^8^ CFU ml^−1^ in sterile 0.8% NaCl) was spread inoculated on Müeller-Hinton (Sigma) agar medium or on KB for strain K, and plastic Etest strips were dispensed onto the inoculated agar surface. After 12 to 72 h of incubation at 28°C, the MIC was determined.

For salt tolerance analysis, KB agar amended with increasing concentrations of NaCl at 0, 0.5, 1, 2, 3, 4, 5, 6, 7, and 10% (wt/vol) was used. The pH ranges for bacterial growth were determined by inoculating each isolate onto KB plates adjusted to various pH values. Growth across a range of temperatures (30, 35, 37, 40, and 42°C) in KB liquid medium was recorded every 24 h for 7 days of culture.

For extracellular enzyme production analysis, proteolytic activity (protease) was conducted on LB supplemented with 1% (wt/vol) skim milk (Oxoid). Catalase activity was evaluated by adding 2 drops of 3% H_2_O_2_ to a loopful of fresh cultures on a glass slide. Oxidase activity was determined using oxidase reagent (bioMérieux) according to the manufacturer’s instructions. Hydrolysis of starch was determined using starch (1%, wt/vol) agar plates flooded with Gram’s iodine after 3 days of incubation. The cellulolytic activities of the isolates were measured by an agar spot method (110).

API (Appareils et Procédés d'Identification; bioMérieux) 50 CH galleries and HiCarbohydrate kit (KB009; HiMedia) were used to test the ability of bacterial isolates to metabolize various substrates as sole carbon sources. Bacterial inoculation was conducted according to the manufacturer’s instructions. Strips were covered and incubated at 28°C. Sugar fermentation results in the formation of acid, which is expressed as a change in color of the indicator from red to yellow. Esculin hydrolysis was indicated to be positive with a color change to black. For the HiMedia kit, citration and malonate utilization was considered positive with a color change to blue.

Activities of constitutive enzymes and other physiological features of the bacteria were generated using the API 20E and API 20NE (bioMérieux). The activities of the 19 enzymes involved in the main nutrient biogeochemical cycles: carbon (β-glucosidase, α-glucosidase, α-galactosidase, β-galactosidase, β-glucuronidase, α-mannosidase, α-fucosidase, esterase lipase, and lipase activities), phosphorous (acid and alkaline phosphomonoesterase and naphthol-AS-BI-phosphohydrolase activities), and nitrogen (protease, leucine-arylamidase, valine arylamidase, and N-acetyl-glucosaminidase activities), were tested according to the API ZYM assay. All API tests were conducted according to the manufacturer’s instructions.

Aerotolerance of selected bacteria was determined in thioglycolate broth (Sigma).

### Protection against phytopathogens by rhizobacteria associated with *Arabidopsis*.

The potential of the four isolated bacteria in reducing disease severity of phytopathogens on *A. thaliana* was investigated, according to Ingle and Roden (111). Arabidopsis thaliana seeds treated with bacterial suspension were plated on one-half-strength Murashige and Skoog (1/2 MS) (pH 5.7) supplemented with 0.8% (wt/vol) agar (*in vitro*) or sown in moistened Jiffy-7 peat pellets (soil assay). For the control, seeds were treated with sterilized 10 mM MgSO_4_. Plants were grown in a growth chamber (Conviron PGR15; Winnipeg, MB, Canada) maintained at 22°C with a 16-h photoperiod with 200 μmol m^−2^ s^−1^ photosynthetic photon flux density at plant height and 60% relative humidity. Each experiment was carried out three times, with comparable results.

For the bacterial pathogenesis assay, an overnight culture of P. syringae pv. tomato DC3000 was subcultured and grown at 28°C until the optical density at 600 nm (OD_600_) reached 0.8. Bacteria were centrifuged (8,000 × *g*, 10 min) and resuspended in 10 mM MgSO_4_. For soil assay, 4-week-old plants were pressure infiltrated on leaves with a P. syringae pv. tomato inoculum (10^6^ CFU ml^−1^) containing 0.004% (vol/vol) Silwer L-77. Mock inoculation (control) was performed with a solution of 10 mM MgSO_4_. At 1 h after challenge inoculation on day 4, bacterial populations in infected leaves were determined. Inoculated leaves from three plants were pooled, weighed, surface sterilized, and homogenized in 0.8% NaCl. To determine the titer of bacterial growth, a dilution series from 10^−1^ to 10^−4^ was plated individually on LB agar plates containing appropriate antibiotics. Colonies were counted after 2 days of incubation at 28°C, and the number of CFU per milligram of infected leaf tissue was determined. For *in vitro* assay, 14-day-old seedlings were inoculated with 2 μl of P. syringae pv. tomato suspension (10^9^ CFU ml^−1^) in the center of the rosette. Seven days after inoculation, colonization levels of P. syringae pv. tomato were determined as the number of CFU per milligram of infected leaf tissue.

For fungus pathogenesis analysis, *Botrytis cinerea* B191 was reactivated on PDA medium amended with streptomycin (20 mg liter^−1^) at 22°C and a 12-h photoperiod. Spores were harvested in sterile water from a culture grown for 10 days and resuspended in potato dextrose broth (PDB) to a spore density of 10^5^ CFU ml^−1^. Fully developed leaves of 4-week-old *Arabidopsis* plants grown in peat pellets were drop inoculated with 5 μl of *B. cinerea* spore suspension. Twelve plants for each treatment were incubated at 22°C. Four days postchallenge, the disease level was assessed by measuring the necrosis diameter induced by *B. cinerea* spores. *In planta* fungal growth was examined by simultaneous analysis of the transcript levels of the *B. cinerea* actin gene (*BcActin*) with primers BcActin-1F (5′-TCCAAGCGTGGTATTCTTACCC-3′) and BcActin-1R (5′-TGGTGCTACACGAAGTTCGTTG-3′) and the *Arabidopsis* actin gene (*AtActin2*), an internal control, using primers AtActin2-1F (5′-GGCGATGAAGCTCAATCCAAACG-3′) and AtActin2-Ar (5′-GGTCACGACCAGCAAGATCAAGACG-3′) (38). Total RNA extracted from *Arabidopsis* leaves and cDNA synthesis was conducted according to Nie et al. (38). Relative fungal growth was determined by ratios of BcActin to AtActin.

### *In vitro* inoculation of maize.

Maize seeds (var. 19K19) were surface sterilized, incubated in bacterial suspension or 10 mM MgSO_4_ for 4 h, and then germinated in petri plates containing 1.5% (vol/wt) agar in water for 4 days in the dark, after which the germinated seeds were transferred aseptically to glass tubes (20- by 2.5-cm diameter) filled with 25 ml of 1/2 Hoagland solution (HS) supplemented, or not, with 100 mM NaCl and maintained at 25°C in a growth chamber under a 16-h photoperiod with a 350-μmol m^−2^ s^−1^ photon flux density. Each endophyte was tested in 12 replicate tubes, randomly distributed in the growth chamber. The experiment was repeated twice. After 3 weeks, plants were removed from the tubes and allowed to air dry for 20 min. Roots and shoots were then dissected from each plant for physiological index measurements.

### Statistical analysis.

For all experiments, the overall data were analyzed by one-way analysis of variance (ANOVA), and differences between control and bacterium treatments were considered statistically significant at the *P ≤ *0.05 level using Tukey’s honestly significant differences (HSD) test of the COSTAT statistical software (CoHort Software, Monterey, CA, USA). Standard errors were calculated for all mean values.

### Data availability.

Genome accession numbers in GenBank for the fragments of *nifH* gene from strains n, L, and Y are MW467562, MW467563, and MW475351, respectively.
