# Supplementary material for: Characterization of Selected Plant Growth-Promoting Rhizobacteria and Their Non-Host Growth Promotion Effects
Source: Microbiol Spectr. 2021 Jun 30;9(1):10.1128/spectrum.00279-21. doi: 10.1128/spectrum.00279-21 (PMC8552778; doi:10.1128/spectrum.00279-21)
Supplement: SUPPLEMENTAL FILE 1 — Download SPECTRUM00279-21_Supp_1_seq2.docx, DOCX file, 2.3 MB [file spectrum00279-21_supp_1_seq2.docx]

| **TABLE S1.** | Primer sets used for real-time PCR in the present study |
| --- | --- |

| **AGI number** | **Gene** | **Primer sequences (5’-3’)** | **Reference** | **Amplicon size (bp)** |
| --- | --- | --- | --- | --- |
| At2g28390 | *SAND* | CAGACAAGGCGATGGCGATA | Chen et al. (2016) | *244* |
|  |  | GCTTTCTCTCAAGGGTTTCTGGGT |  |  |
| At5g44420 | *PDF1.2* | CTTGTTCTCTTTGCTGCTTTCGAC | current study | *106* |
|  |  | ATGCATTACTGTTTCCGCAAACC |  |  |
| At1g15550 | *GA3ox1* | CCAAATCTCAAACCACGGCG | current study | *81* |
|  |  | ACAGGTAGCCCGAAGAGACT |  |  |
| At3g45640 | *MPK3* | TGACGTTTGACCCCAACAGA | Kim et al. (2014) | *146* |
|  |  | CTGTTCCTCATCCAGAGGCTG |  |  |
| At2g43790 | *MPK6* | CCGACAGTGCATCCTTTAGCT | Kim et al. (2014) | *92* |
|  |  | TGGGCCAATGCGTCTAAAAC |  |  |
| At2g39800 | *P5CS1* | GTGCTAGATGGAATCTGTCATGT | current study | *108* |
|  |  | CGCATTACAGGCTGCTGGAT |  |  |
| At2g14610 | *PR1* | ATAATCAGTTGCAACTATGATCCTC | Kim et al. (2014) | *200* |
|  |  | AAATAGATTCTCGTAATCTCAGCTC |  |  |
| At4g36110 | *SAUR9* | GCGTTGTCTCAAGCAGCATC | current study | *140* |
|  |  | TAGCGACTTCGGTGTTGACC |  |  |
| At3g26830 | *PAD3* | GTGGAGTCGCTGGCATAACA | current study | *101* |
|  |  | GTCCCCAAGTGTTGTCCGAA |  |  |
| At5g52310 | *RD29A* | ACAAAACACACATAAACATCCAAAGT | Kim et al. (2014) | *98* |
|  |  | ATCACTTGGCTGCACTGTTGTTC |  |  |
| At5g42300 | *RD29B* | GAATCAAAAGCTGGGATGGA | Kim et al. (2014) | *197* |
|  |  | TGCTCTGTGTAGGTGCTTGG |  |  |

**REFERENCES**

Chen X, Yuan L, Ludewig U. 2016. Natural genetic variation of seed micronutrients of *Arabidopsis thaliana* grown in zinc-deficient and zinc-amended soil. Front Plant Sci 7: 1070.

Kim K, Jang YJ, Lee SM, O BT, Chae JC, Lee KJ. 2014. Alleviation of salt stress by *Enterobacter sp.* EJ01 in tomato and Arabidopsis is accompanied by up-regulation of conserved salinity responsive factors in plants. Mol Cells 37: 109-117.

| **TABLE S2.** | Some phenotypic characteristics of isolated rhizobacteria |
| --- | --- |

| **Characteristic** | **Strains** |  |  |  |
| --- | --- | --- | --- | --- |
|  | **n** | **L** | **K** | **Y** |
| Colony color on KB | yellow | milk white | light pink | white |
| Grams’ reaction | N | P | N | N |
| Voges proskauer | + | + | + | − |
| Motility | + | + | − | + |
| Oxygen requirement | obligate aerobe | facultative anaerobe | aerobe | microaerophiles |
| Catalase | + | + | w | + |
| Oxidase | − | − | − | − |
| Urease | − | − | − | + |
| Fermentation/oxidation (glucose) | ? | − | − | − |
| Nitrates reduction to nitrites | − | + | − | − |
| Arginine dihydrolase | + | + | − | − |
| Tryptophane deaminase | + | + | + | − |
| **Hydrolysis of:** | | | | |
| Starch (amylase) | − | + | − | − |
| CMC (cellulase) | − | + | − | + |
| Casein (protease) | + | + | + | − |
| Gelatin (gelatinase) | − | + | − | − |
| **Growth tolerance:** |  | | | |
| 37 °C | + | + | − | + |
| 40 °C | − | + | − | − |
| NaCl | 6% | 7% | 1.2% | 1.5% |
| pH range | 5.0-10.0 | 5.0-10.0 | 5.0-8.0 | 5.0-8.0 |
| **Enzyme activities (API ZYM):** | | | | |
| Alkaline phosphatase | − | + | + | − |
| Esterase lipase | + | + | − | − |
| Leucine-arylamidase | + | + | + | + |
| Valine arylamidase | + | − | + | − |
| Cystin arylamidase | − | − | + | − |
| Acid phosphatase | − | + | + | + |
| Naphtol-AS-BI-phosphohydrolase | + | − | − | − |
| α-galactosidase | − | − | + | − |
| β-galactosidase | − | − | + | + |
| α-glucosidase | − | − | + | + |
| β-glucosidase | − | + | + | + |
| N-acetyl-β-glucosaminidase | − | − | + | − |
| α-fucosidase | − | − | + | − |

+: indicates a positive reaction (color change due to substrates consumption) while − indicates a negative reaction. N: negative Gram reaction; P: positive Gram reaction.

| **TABLE S3.** | Carbon source utilization by isolated rhizobacteria *^a^* |
| --- | --- |

| **Acid fermentation** | **Strains** |  |  |  |
| --- | --- | --- | --- | --- |
|  | **n** | **L** | **K** | **Y** |
| Glycerol | + | − | − | − |
| L-Arabinose | + | − | − | + |
| D-Xylose | + | − | − | + |
| D-Galactose | + | − | + | + |
| D-Glucose | + | + | + | − |
| D-Fructose | + | + | + | + |
| D-Mannose | + | − | + | − |
| D-Mannitol | + | − | − | + |
| Arbutin | − | + | − | − |
| Esculin hydrolysis | − | + | + | + |
| Salicin | − | + | − | − |
| Maltose | − | + | + | − |
| Trehalose | + | + | + | − |
| Adonitol | − | − | − | + |
| Erythritol | − | − | − | + |
| Starch | − | + | − | − |
| Glycogen | − | + | − | − |
| D-Fucose | + | − | − | + |
| Ribose | + | + | − | − |
| N-Acetylglucosamine | − | + | − | − |
| Melibiose | + | − | + | − |
| Sucrose | − | − | + | − |
| D-Cellobiose | − | − | + | − |
| Caprate | + | − | − | − |
| Adipate | + | + | − | + |
| Maliate | + | + | − | + |
| Phenyl-acetate | − | + | − | − |
| Citrate utlization | + | + | − | + |
| Dextrose | + | + | + | − |
| Raffinose | − | − | + | − |
| Malonate utilization | + | + | − | + |

*^a^* This experiment was tested in API CH strips and HiMedia kits, respectively. The color change was observed after 1-3 days of incubation at 28 °C.

+: positive result corresponds to acidification revealed by the phenol red indicator changing to yellow.

| **TABLE S4.** | Susceptibility (MICs) of bacterial isolates to various antibiotics (μg mL^-1^) as determined by Etest method *^a^* |
| --- | --- |

| **Isolate** | **Treatment** | | | |
| --- | --- | --- | --- | --- |
|  | **n** | **L** | **K** | **Y** |
| Ampicillin | >256 | >256 | >256 | 0.064 |
| Erythromycin | >256 | 3 | 24 | 0.5 |
| Kanamycin | 1.5 | 2.5 | >256 | 0.38 |
| Chloramphenicol | >256 | 2 | 16 | 12 |
| Gentamicin | 0.38 | 0.38 | 16 | 0.125 |
| Streptomycin | 3 | 1.5 | 48 | 8 |
| Tetracycline | 3 | 0.38 | 16 | 0.75 |
| Rifampicin | 1.5 | 0.38 | >32 | 8 |
| Vancomycin | >256 | 2 | 24 | >256 |
| Tobramycin | 1.0 | 1.5 | >256 | 0.125 |

*^a^* Experiments were repeated three times with same results.


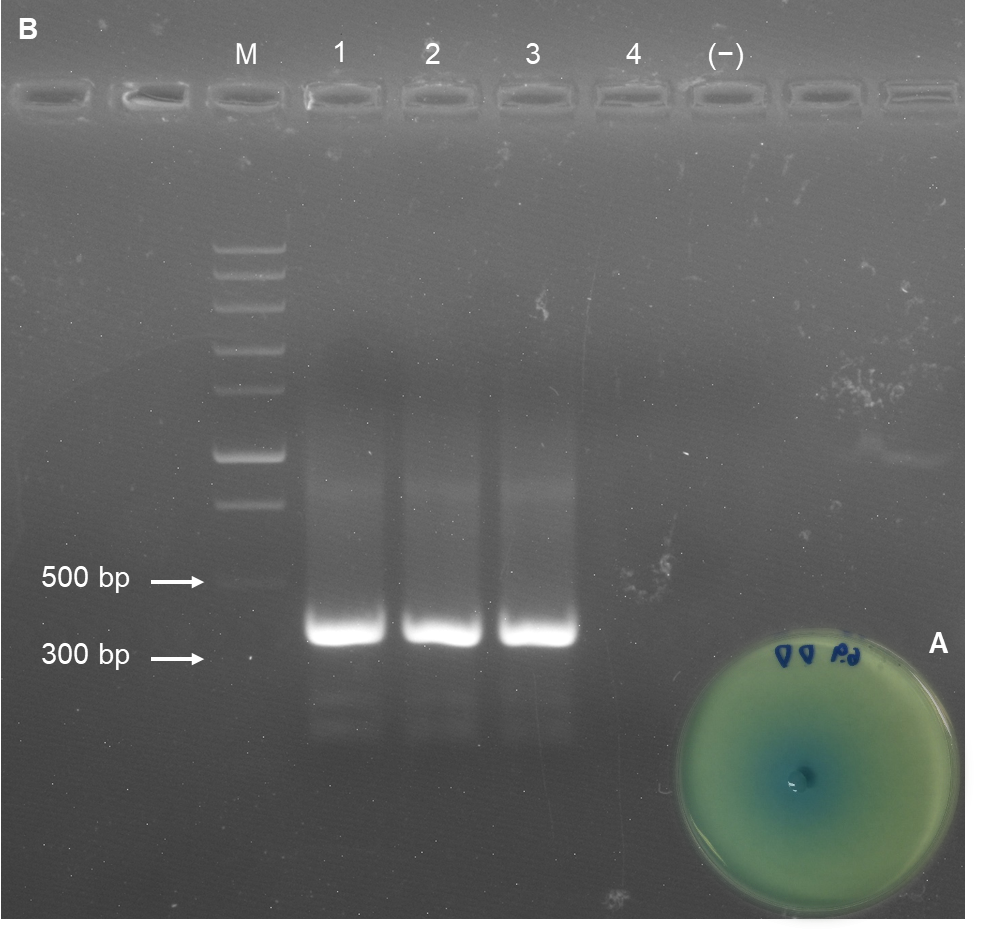


**FIGURE S1** Nitrogen-fixing capacity of selected strains as determined by (a) Nfb solid medium (inset; strain Y) and (b) PCR-amplification using primer pairs Ueda19F and 388R of *nifH* gene from genomic DNA of selected strains. Electrophoretic patterns of PCR products of the *nifH* gene showed that 3 out of the 4 selected strains harbor the nitrogen-fixing gene. Lanes: 1-4, n, L, Y and K respectively; (−), negative control (water); M, Fast DNA Ladder.


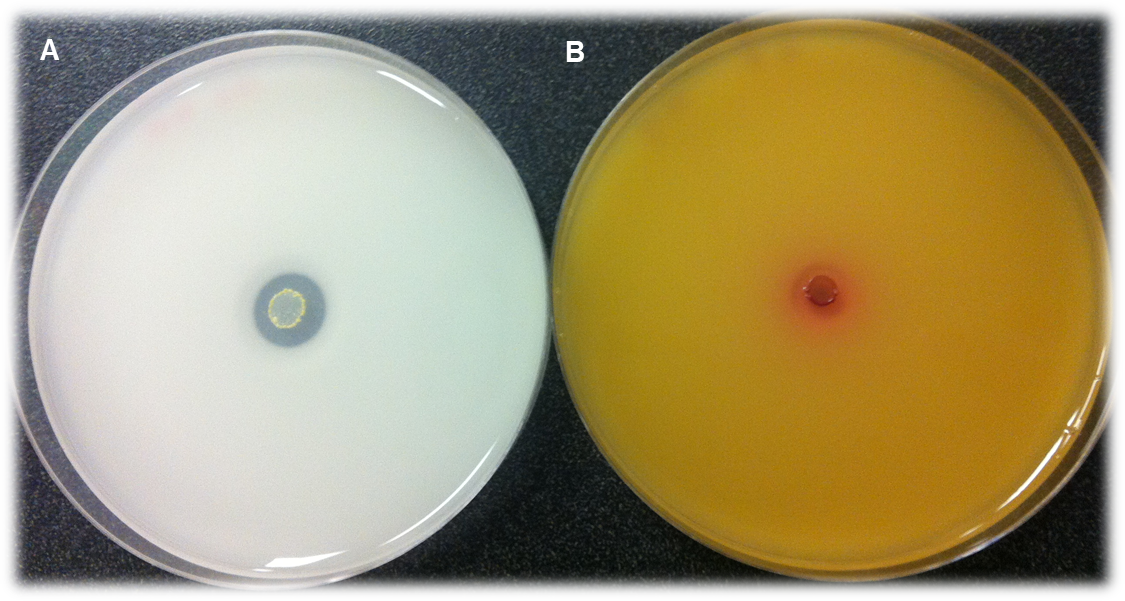


**FIGURE S2** Representative P-solubilizing phenotype of strain n on different screening media. (a) NBRIP agar (b) Buffered medium. The ability to solubilize and use Ca_3_(PO_4_)_2_ as a sole source of phosphate was evident as a clearing zone around the colony of strain n.


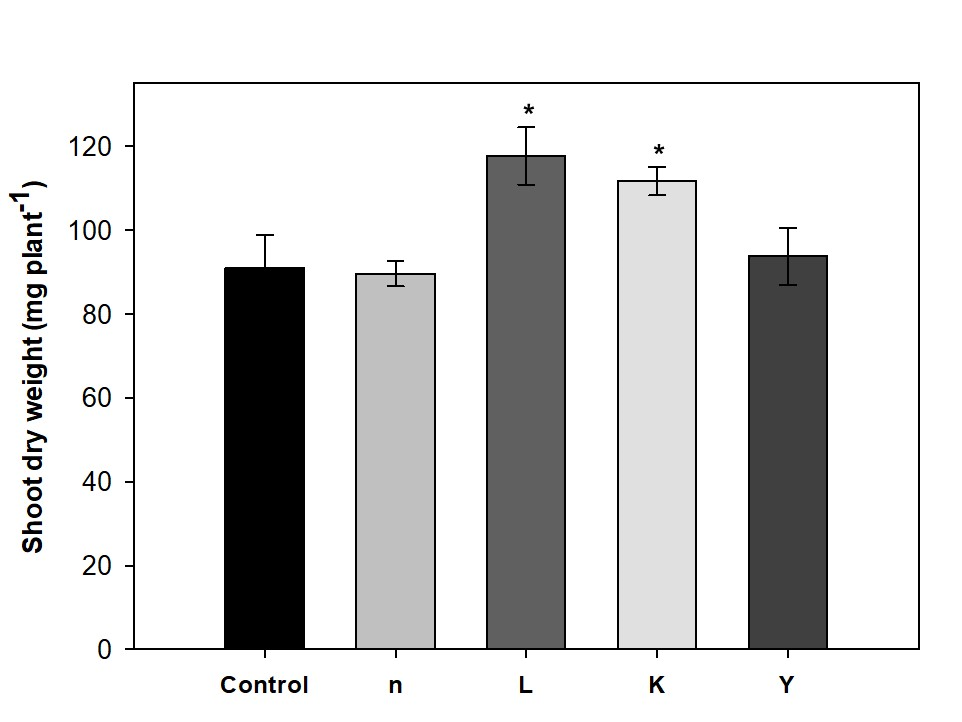


**FIGURE S3** Effects of seed-treatment of maize with selected rhizobacteria on shoot dry weight at D21. Asterisks (*) indicates statistically significant differences from control plants (P < 0.05).
